# Supplementary material for: Are All Prognostic Stage IB Breast Cancers Equivalent?
Source: Cancers (Basel). 2024 Nov 14;16(22):3830. doi: 10.3390/cancers16223830 (PMC11592610; doi:10.3390/cancers16223830)
Supplement: Supplementary file 1 [file cancers-16-03830-s001.zip › cancers-3290404-supplementary.pdf]

cancers MDPI

Supplemental Material

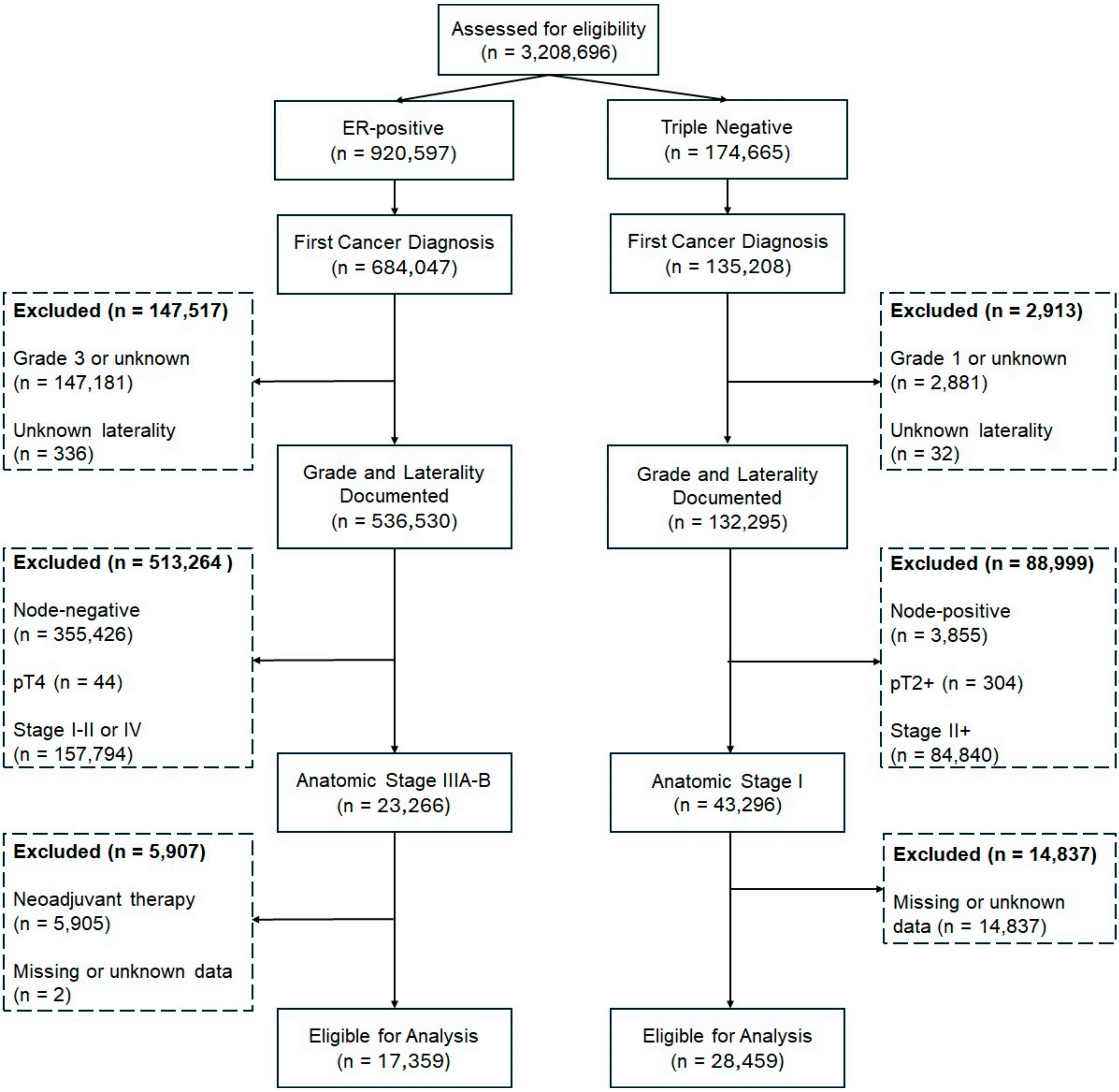

Figure S1. CONSORT Diagram.

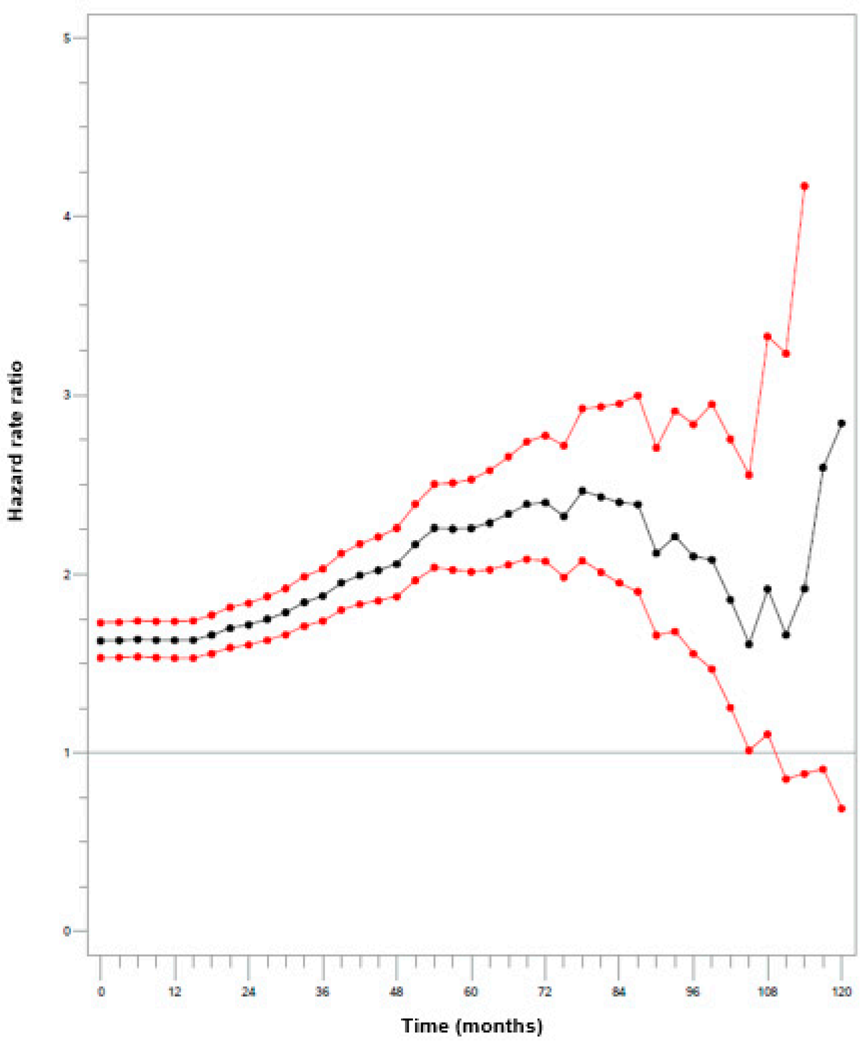

Figure S2. Landmark analysis demonstrating change in hazard ratio over time (months) for the entire cohort.

Table S1. Multivariable analysis for entire cohort, regardless of treatment.

	HR (95% CI)	p-value
LA-HR+/HER2- vs. ES-TNBC	1.63 (1.53-1.73)	<0.0001*
Age		
<40 yrs	1.04 (0.85-1.26)	0.720
40-49 yrs	0.84 (0.74-0.94)	0.003*
50-59 yrs	Reference	Reference
60-69 yrs	1.06 (0.96-1.17)	0.247
≥70 yrs	2.30 (2.08-2.54)	<0.0001*
1+ v 0 comorbidities	1.59 (1.49-1.70)	<0.0001
Race/Ethnicity		
White	Reference	Reference
Black	1.16 (1.07-1.27)	0.001*
Hispanic^	0.68 (0.58-0.80)	<0.0001*
Asian	0.60 (0.47-0.77)	<0.0001*
Other	0.82 (0.53-1.26)	0.367
Unknown Race/Ethnicity	1.06 (0.91-1.25)	0.447
Insurance Status		
Private insurance	Reference	Reference
Government insurance	1.77 (1.63-1.92)	<0.0001*
No insurance	1.36 (1.06-1.75)	0.017*
Unknown insurance	1.32 (0.97-1.80)	0.076
Median income		
≥$63K/year	Reference	Reference
<$63K/year	1.18 (1.09-1.28)	<0.0001*
Unknown	1.29 (0.32-5.16)	0.720
Facility Type		
Academic	Reference	Reference
Non-academic	1.13 (1.06-1.22)	0.001*
Unknown	-	-

LA-HR+/HER2- - Locally advanced hormone receptor positive (including estrogen and progesterone receptor positive) and HER2 negative; TNBC-triple negative breast cancer; *Statistically significant with $p < 0.05$

^ - All other racial categories not listed as "Hispanic" are specifically coded as non-Hispanic

Table S2. Multivariable analysis for entire cohort included in the landmark analysis.

	HR (95% CI)	p-value
LA-HR+/HER2- vs. ES-TNBC	2.26 (2.04-2.50)	<0.0001*
Age		
<40 yrs	1.04 (0.76-1.42)	0.801
40-49 yrs	0.75 (0.61-0.91)	0.0042*
50-59 yrs	Reference	Reference
60-69 yrs	1.02 (0.86-1.20)	0.846
≥70 yrs	2.55 (2.16-3.01)	<0.0001*
1+ v 0 comorbidities	1.58 (1.33-1.89)	<.0001
Race/Ethnicity		
White	Reference	Reference
Black	1.13 (0.97-1.31)	0.115
Hispanic^	0.56 (0.41-0.76)	0.0002*
Asian	0.50 (0.32-0.78)	0.002*
Other	0.25 (0.06-1.01)	0.052
Unknown Race/Ethnicity	1.03 (0.81-1.32)	0.798
Insurance Status		
Private insurance	Reference	Reference
Government insurance	1.69 (1.47-1.93)	<0.0001*
No insurance	1.71 (1.18-2.48)	0.005*
Unknown insurance	1.42 (0.87-3.00)	0.159
Median income		
≥$63K/year	Reference	Reference
<$63K/year	1.16 (1.02-1.33)	0.024*
Unknown	1.78 (0.25-12.65)	0.566
Facility Type		
Academic	Reference	Reference
Non-academic	1.07 (0.95-1.20)	0.284
Unknown	-	-

LA-HR+/HER2- - Locally advanced hormone receptor positive (including estrogen and progesterone receptor positive) and HER2 negative; TNBC - triple negative breast cancer; *Statistically significant with $p < 0.05$

^ - All other racial categories not listed as "Hispanic" are specifically coded as non-Hispanic
